# Selective Bacteriocins: A Promising Treatment for *Staphylococcus aureus* Skin Infections Reveals Insights into Resistant Mutants, Vancomycin Resistance, and Cell Wall Alterations

**DOI:** 10.3390/antibiotics12060947

**Published:** 2023-05-23

**Authors:** Félix Jaumaux, Kenny Petit, Anandi Martin, Hector Rodriguez-Villalobos, Marjorie Vermeersch, David Perez-Morga, Philippe Gabant

**Affiliations:** 1 Structure et Fonction des Membranes Biologiques (SFMB), ULB-Campus Plaine Building BC 3rd Floor Wing C, Blvd Triomphe Access 2, 1050 Brussels, Belgium; 2 Syngulon, 1402 Seraing, Belgium; 3 Cliniques Universitaires Saint-Luc, Avenue Hippocrate 10, 1200 Brussels, Belgium; 4 Center for Microscopy and Molecular Imaging (CMMI), Université Libre de Bruxelles, Campus de Charleroi—Gosselies (Biopark)—CP 300, Rue Prof. Jeener & Brachet, 12, 6041 Gosselies, Belgium; 5 Laboratoire de Parasitologie Moléculaire, Université Libre de Bruxelles, Campus de Charleroi—Gosselies (Biopark)—CP 300, Rue Prof. Jeener & Brachet, 12, 6041 Gosselies, Belgium

**Keywords:** antibiotic resistance, *S. aureus*, antimicrobial peptides, bacteriocins, skin microbiome, selective inhibition, *walK/R* two-component system, vancomycin, cell wall thickening

## Abstract

The emergence of antibiotic-resistant *S. aureus* has become a major public health concern, necessitating the discovery of new antimicrobial compounds. Given that the skin microbiome plays a critical role in the host defence against pathogens, the development of therapies that target the interactions between commensal bacteria and pathogens in the skin microbiome offers a promising approach. Here, we report the discovery of two bacteriocins, cerein 7B and cerein B4080, that selectively inhibit *S. aureus* without affecting *S. epidermidis*, a commensal bacterium on the skin. Our study revealed that exposure of *S. aureus* to these bacteriocins resulted in mutations in the *walK/R* two-component system, leading to a thickening of the cell wall visible by transmission electron microscopy and subsequent decreased sensitivity to vancomycin. Our findings prompt a nuanced discussion of the potential of those bacteriocins for selective targeting of *S. aureus* on the skin, given the emergence of resistance and co-resistance with vancomycin. The idea put forward implies that by preserving commensal bacteria, selective compounds could limit the emergence of resistance in pathogenic cells by promoting competition with remaining commensal bacteria, ultimately reducing chronical infections and limiting the spread of antibiotic resistance.

## 1. Introduction

On a global scale, *Staphylococcus aureus* is the second killer when it comes to deaths associated with antibiotic resistance [1]. *S. aureus* is responsible for various infections such as skin abscesses, wound infections, deep tissue abscesses, osteomyelitis, endocarditis, toxic shock syndrome, sepsis, and bacteremia [2]. This Gram-positive bacterium possesses several mechanisms to reduce its sensitivity to antibiotics, including the formation of biofilms, small colonies variants [3], the use of efflux pumps [4], persistence, and the ability to infiltrate human cells [5]. This pathogen first caused issues due to the emergence of methicillin-resistant *Staphylococcus aureus* (MRSA). With vancomycin used as a key antibiotic in the treatment of severe bacteremia caused by this pathogen, the emergence of vancomycin-intermediate *Staphylococcus aureus* (VISA) and vancomycin-resistant *Staphylococcus aureus* (VRSA) strains (defined has having MIC > 4 μg/mL and MIC ≥ 16 μg/mL respectively) is a major concern [6].

In recent years, the microbiome has emerged as an important element for disease prevention and new treatment development. Because serious *S. aureus* infections first start as the skin is breached, understanding the skin microbiome is relevant [7]. One commensal bacterium present on the skin, *Staphylococcus epidermidis*, seems to be the best candidate to compete with *S. aureus* due to its positive interaction with the immune system [8], its inhibition of *S. aureus* biofilm formation through the production of a serine protease [9], and the growth inhibition of *S. aureus* by the production of bacteriocins [10]. Those properties make the discovery and development of selective antimicrobial compounds that do not inhibit the growth of *S. epidermidis* promising for skin microbiome treatments aimed at restoring balance to the skin microbiome in situations of dysbiosis caused by *S. aureus* colonization.

Bacteriocins are ribosomally synthesized antimicrobial peptides (AMPs) produced by bacteria that have the advantage of having a specific spectrum of action, making them a proposed alternative to conventional antibiotics for the treatment of bacterial infections. It has been previously shown that several bacteriocins can inhibit the growth of *S. aureus*. Examples of such bacteriocins are the nisins, a well-studied group of bacteriocins that binds to lipid II, permeabilizes the cell membrane, and inhibits cell wall synthesis [11]. Another example is the mutacins produced by *Streptococcus mutans*, including mutacin III, I, IIIb, B-Ny266, and IVb, which are also active against *S. aureus* [12]. In addition, it was shown that mice infected with *S. aureus* survived at a higher rate when treated with mutacin B-Ny266 compared to the infected control group, with an effective dose protecting 50% of the animals (ED50) found to be comparable to that of vancomycin [13].

Those bacteriocins have various mechanisms of action, including targeting the cell membrane through pore formation or specific binding to lipids or membrane proteins [14]. Some bacteriocins do require the presence of a specific protein receptor on the cell membrane for activity; examples are the maltose ATP-binding cassette (ABC) transporter, which was shown to be the receptor for garvicin ML [15], and the mannose phosphotransferase system (Man-PTS), which was reported as a receptor for various bacteriocins, such as bacteriocins from the pediocin group, the lactococcin A, leucocin A, pediocin PA-1, enterocin, lactococcin B, and the garvicin Q [16,17]. Understanding the selectivity of those bacteriocins remains complex and not solely determined by the presence or absence of a receptor, as the amino acid sequence of the receptor also plays a role. The mechanism of action of pore-forming bacteriocins includes the barrel-stave, toroidal pore, and carpet models, with all models beginning with the binding of peptides to the membrane surface, causing stretching and thinning of the membrane at the binding site, leading to pore formation [18]. Additionally, some bacteriocins can enter the inside of the cell and inhibit essential functions leading to cell death [19].

The ability of *S. aureus* to rapidly adapt to new environmental conditions underscores the need for caution when employing any new antimicrobial agents. Therefore, it is important to consider the potential impact of bacteriocins on bacterial resistance when developing new treatments.

We propose to explore the use of bacteriocins for their applications in skin treatments for *S. aureus* infections using PARAGEN [20], a centralized collection of bacteriocin built by Syngulon to enable fast screening.

## 2. Results

### 2.1. Discovery of Selective Bacteriocins

**Figure 1:**
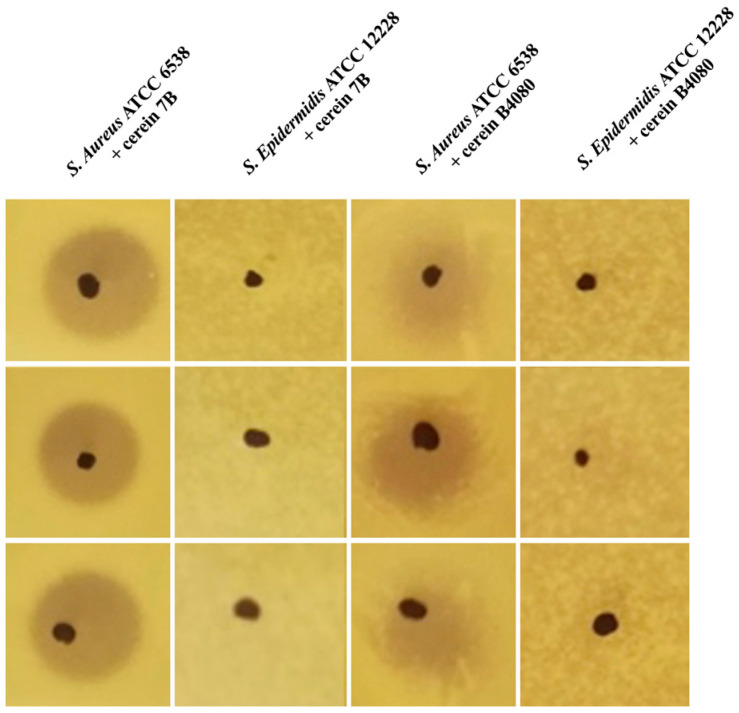
Spot assays realized on *S. aureus* and *S. epidermidis* using the cerein 7B and cerein B4080. The figure depicts the spot assays results, where the presence of inhibition on *S. aureus* and the absence of inhibition on *S. epidermidis* confirm the selectivity of both the cerein 7 and cerein B4080.

The PARAGEN collection comprises various bacteriocins which, due to their short length (<60 amino acids) and lack of post translational modifications, can be synthesized chemically. Those bacteriocins from the collection were used to realize as fast screening performed on *S. aureus* ATCC 6538 and on *S. epidermidis* ATCC 12228 by spot assays. As illustrated in Figure 1, the presence of an inhibition halo on *S. aureus* but not on *S. epidermidis* allowed for the identification of two bacteriocins which possess activities against *S. aureus*, with no effect against *S. epidermidis*: cerein 7B, a 56 amino acid peptide, previously identified as a product of *Bacillus cereus* Bc7 [21]; and cerein B4080, a leaderless bacteriocin, which was formerly identified after an in silico analysis and added to the PARAGEN collection [20] as lacticin Z-Variant 2 (novel). However, due to the fact that the gene coding for this bacteriocin sequence was discovered in *Bacillus cereus* B4080, we have renamed this bacteriocin as cerein B4080.

We evaluated those bacteriocins’ activities against multiple bacterial strains by spot assays, including *Lactococcus lactis* IL1403, for which both bacteriocins were found to be active and *Escherichia coli* MG 1655 for which no activity was observed. Neither bacteriocin demonstrated activity against *Propionibacterium acnes* ATCC 6919, a bacterium associated with the development of acne [22], while cerein B4080 was the only bacteriocin of the two to be found active against the human commensal *Staphylococcus hominis* ATCC 27844, Table 1.

**Figure 2:**
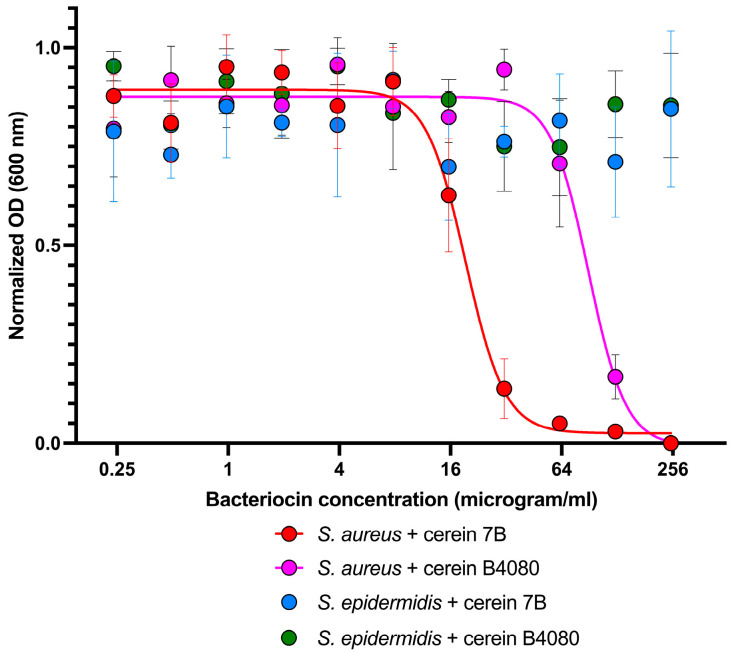
The determination of the minimum inhibitory concentration (MIC) of cerein 7B and cerein B4080 against *S. aureus* ATCC 6538. Our results show that the MIC50 value of cerein 7B against *S. aureus* ATCC 6538 is 20 micrograms/mL, and the MIC value of the cerein B4080 was determined to be 90 µg/mL. No significant impact on the growth of *S. epidermidis* was observed, even at the highest tested concentration of 250 micrograms/mL of cerein 7B or of cerein B4080.

This observed selectivity in the spot assay was then confirmed by the evaluation of the minimum inhibitory concentration (MIC) against *S. aureus* ATCC 6538 and *S. epidermidis* ATCC 12228 using liquid dilution assays (in Figure 2). Where the MIC value for cerein 7B against *S. aureus* was determined to be 20 µg/mL and the MIC value of the cerein B4080 was determined to be 90 µg/mL, no growth inhibition was observed for *S. epidermidis* at concentrations up to 250 µg/mL.

### 2.2. Cerein 7B, a Membrane Anchoring Bacteriocin

Numerous bacteriocins have been reported to exert destabilizing effects on the cellular membrane, either by inducing membrane destabilization or by forming pores into the membrane. We decided to focus on the cerein 7B. To gain insights into its mechanism of action, we utilized AlphaFold, a powerful protein structure prediction tool, developed by DeepMind, an artificial intelligence research laboratory headquartered in the United Kingdom, to perform a structural prediction of cerein 7B. The resulting structural model revealed that cerein 7B consists of two parallel alpha helices linked by a loop containing a proline residue.

**Figure 3:**
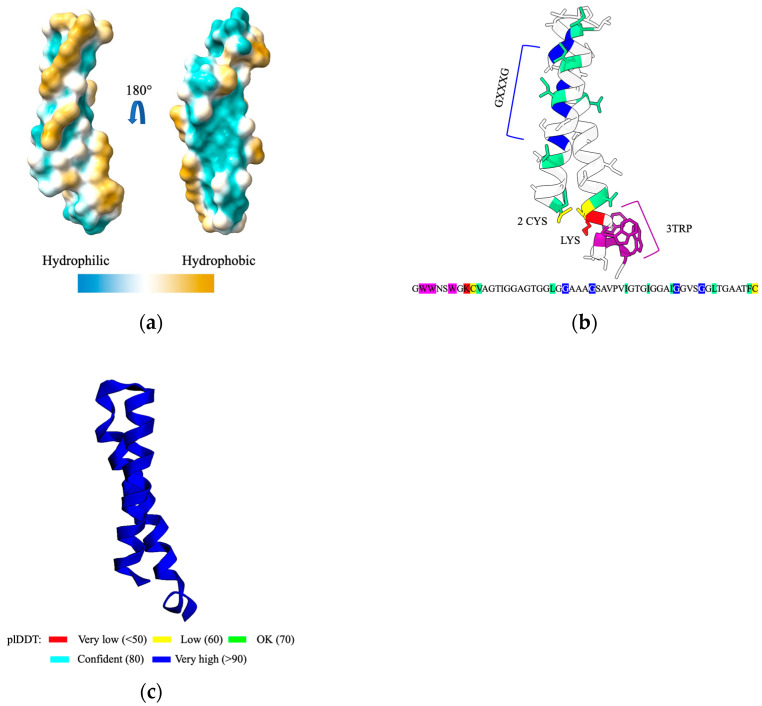
This figure depicts the predicted structure of cerein 7B, generated using AlphaFold: (**a**) shows the hydrophobic surface of the bacteriocin; (**b**) highlights the importance of various amino acids in cerein 7B that was gained through the alanine scanning. The critical GXXXG motifs are colored blue, while the two cysteines required for the protein’s activity are colored yellow. The lysine residue, the only charged amino acid in the sequence, is highlighted in red, and the three tryptophan residues are marked in magenta. (**c**) shows the predicted pIDDT of the cerein 7B for the best obtained model.

To explore the structure–function relationship of cerein 7B in greater depth, we conducted an alanine scanning of the entire peptide. Both the wild type cerein 7B and all mutated versions were expressed in vitro, and their activities were assessed by spot assays on both *S. aureus* ATCC 6538 and *L. lactis* ILI403. Most mutations (30/47) did not affect the antimicrobial activity of the bacteriocin on *L. lactis* ILI403. However, 17 mutants showed loss of activity, providing insight into a mutative mode of action (Figure 3). The list of generated mutants and the spot assay results can be found in the Supplementary Material Figure S2 and Table S1.

In particular, K8A, W2A, W3A and W6A were not able to prevent the growth of *S. aureus* in our assay. Both Lysine (through ionic interaction with the phosphate of the lipid headgroup) and tryptophan are membrane-anchoring residues when located at the ends of transmembrane helices [23]. This result thus supports the hypothesis that cerein 7B requires membrane anchoring to be biologically active. Furthermore, two GXXXG motifs in the peptide sequence, specifically G24AAAG28 and G43GVSG47, led to a loss of activity if either of the glycine were replaced by an alanine. These motifs are known facilitated helix–helix interactions and can lead to dimerization [24], suggesting that cerein 7B alpha helices interact together to exert their antimicrobial activity. Additionally, numerous hydrophobic amino acids showed a loss of activity, some of which forming a hydrophobic region on the bacteriocin. This hydrophobic region likely then interacts with the hydrophobic region of the lipid bilayer that makes up the membrane.

Taken together, our findings suggest that cerein 7B likely interacts and anchors itself to the bacterial membrane through lysine and tryptophan residues, while the GXXXG motifs and Cysteines play a role in alpha helix stabilization, allowing the peptide to exert its potent antimicrobial activity. However, these results do not indicate whether cerein 7B is active as a monomer or a multimer.

### 2.3. Isolation of Mutants Able to Grow in Presence of Bacteriocins

To anticipate the impact of the selective pressure on *S. aureus* upon treatment using the bacteriocins, colonies able to grow in medium containing 2xMIC were isolated. Exposure to 40 µg/mL of cerein 7B led to a frequency of resistance of one in one million with six mutants, numbered 1 to 6, selected for further characterization. The same processes were realized for the cerein B4080 with 200 µg/mL, where the rate of apparition ended up being two per million and where two mutants were selected for further characterization, numbered named 7 and 9, respectively.

**Figure 4:**
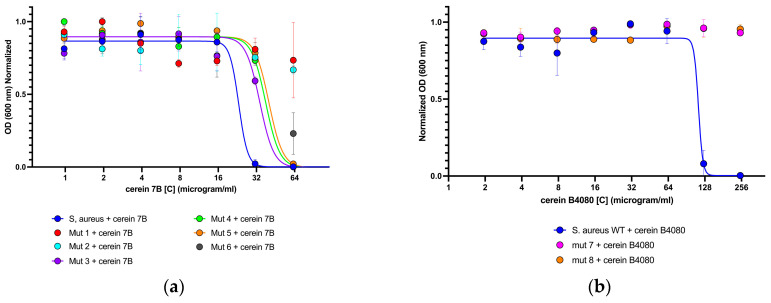
This figure illustrates the difference in sensitivity between *S. aureus* ATCC 6538 and the isolated mutants: (**a**) presents the quantification of the variation in sensitivity to cerein 7B between the wild-type strain of *S. aureus* and the six isolated mutants; (**b**) presents the quantification of the variation in sensitivity to cerein B4080 between the wild-type strain of *S. aureus* and the two isolated mutants.

In order to confirm the reduced sensitivity of the isolated mutants towards the bacteriocin, a growth assay was conducted with the wild type and mutant strains. As shown in Figure 4, the mutant strains are able to grow in the presence of higher concentrations of bacteriocin as compared to the wild type *S. aureus* strain. Specifically, for both bacteriocins, the mutants displayed growth at twice the inhibitory concentration of the wild-type strain. Remarkably, mutant strains 1 and 2 displayed a growth capacity exceeding four-fold the inhibitory concentration of cerein 7B, whereas mutants 7 and 8 exhibited a comparable growth capability of at least four times the inhibitory concentration for cerein B4080.

### 2.4. Resistance-Associated Mutations in Two Component Systems

To gain insights into the underlying mechanism responsible for conferring resistance and decreased sensitivity towards cerein 7B and cerein B4080, we conducted whole-genome sequencing (WGS) to investigate the presence of mutations in the genomes. Those mutations are reported in Table 2. The eight isolated mutants were found to be different from one another, but they all contained mutations in either the histidine kinase *walK* or the response regulator *walR* genes.

These genes are part of the *walK/R* two-component regulatory system, which is also known as *yycFG* or *vicRK* [25]. This TCS plays a crucial role in regulating various cellular processes in *S. aureus*, including virulence regulation, biofilm production, oxidative stress resistance, cell wall synthesis, and metabolic processes [26]. The WalK protein is composed of five domains, which include the extracellular domain, the PAS domain, which acts as a molecular sensor, the phosphor-acceptor domain, responsible for the autophosphorylation of the histidine, the HATPase_c domain, which utilizes the energy provided by ATP hydrolysis, and the HAMP domain, which regulates the phosphorylation of the receptor.

The study reports novel mutations occurring in three of those domains: the G223A was located in the HAMP protein domain, the I303S in the PAS protein domain, and the R376P, as well as the previously known mutation, the V383I in the phosphor-acceptor protein domain. WalR is composed of only two domains, the receiver domain affected by the mutations S105R and A111V, and the transcriptional regulator domain affected by the R222V mutation. It is known from previously published single nucleotide mutations in various protein domains of WalK/R that can lead to a decrease in vancomycin sensitivity [27]. Those mutations are reported to lead to a thickening of the cell wall, causing a decrease in sensitivity due to the peptidoglycan-clogging theory, which postulates that the passage of vancomycin molecules across thickened peptidoglycan layers is hindered and delayed, leading to resistance.

The study reports a novel A55P mutation in the auxiliary protein YycH, which interacts with YycI and the WalK histidine kinase to activate the WalK/R two-component system in *Staphylococcus aureus*, regulating cell wall metabolism. Mutations in yycH and yycI have previously been shown to reduce vancomycin susceptibility in clinical VISA strains [28].

Mutations outside of the genes *walK/R* were also found. Notably, all isolated mutants except for mutant number 3 had the same H228N mutation in the *aroE* gene, which encodes a shikimate dehydrogenase (NADP(+)), an essential protein. Unlike the other mutants, mutant number 3 differed from the others in that it lacked a mutation in the *walk* gene but possessed a mutation in the *walR* gene (A111V). This suggests a possible connection between mutations in *walk* and the *aroE* genes, although this connection remains unclear. Four other mutations were found in other genes: K385I in *tkt*, Y339T in *nikA*, V196L in *SLL5*, and V304M in *menD*. The K385I mutation in *tkt* may affect the pentose phosphate pathway, which generates pentose sugars and NADPH [29]. The Y339T mutation in *nikA* is expected to alter nickel transport [30], and the V304M mutation in *menD* may affect the biosynthesis of menaquinone, a form of vitamin K2. These mutations may have functional consequences on their respective metabolic pathways. An additional gene was discovered to be mutated in mutant 7, coding for *SSL5*, *a Staphylococcal* superantigen-like protein [31].

DNA Differences in between the *S. aureus* ATCC 6538 used in this study and its reference genome are available in Supplementary Material Table S2.

### 2.5. Mutations Leading to Resistance against Cerein7b Are Associated to Decreased Sensitivity to Vancomycin

The presence of mutations in a two-component system being involved in the cell wall regulation and known to be involved in vancomycin resistance [27] led to the hypothesis that the isolated mutants might have gained a decreased sensitivity to vancomycin. Out of the mutations identified in this study, one of them had already been reported in the literature as involved in VISA, the V383I [32], while the others are to our knowledge newly discovered.

We performed an antibiotic sensitivity assay to assess the sensitivity of the mutants to vancomycin, Table 3. Mutants 1, 2, 6, 7, and 8 exhibited decreased sensitivity to vancomycin, with mutants 1 and 6 showing a two-fold change in MIC value and mutant 2 showing a four-fold change. No other variations in antibiotic resistance were observed between the wild type and isolated mutants.

### 2.6. Resistant Mutant Display Cell Wall Thickening

**Figure 5:**
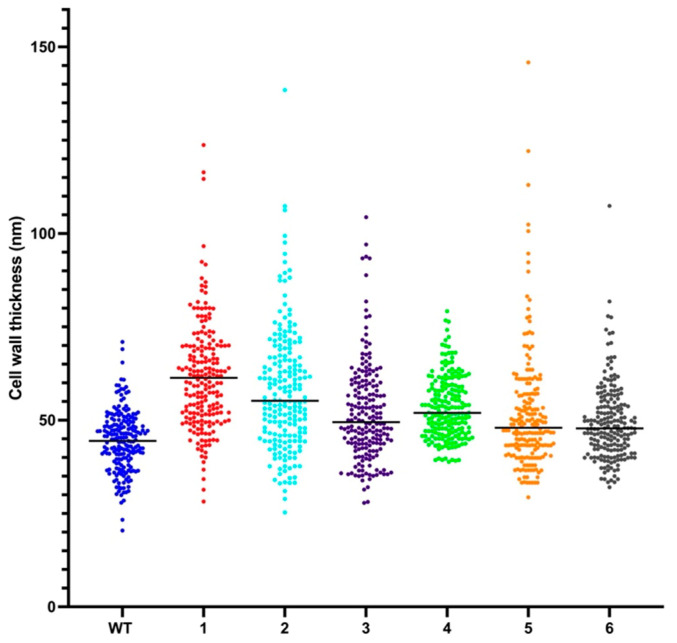
The results of transmission electron microscopy (TEM) measurement of the cell wall thickness in the exponential phase and in the stationary phase for both the wild-type strain of *S. aureus* ATCC 6538 and the six isolated mutants. The TEM images provide insight into the comparison of the cell wall thickness between the strains.

The decrease in sensitivity towards vancomycin in *S. aureus* due to mutations in *WalK/R* has been linked to a cell wall thickening in previous works [33]. To investigate possible changes in cell wall thickness, we used Transmission Electron Microscopy (TEM) on the siz isolated mutants against the cerein 7B. A first set of observations was realized on the wild-type strain and isolated mutant 2 in both the exponential phase and the stationary phase, and showed that a thicker cell wall is present but only at the stationary phase, with an average cell wall thickness of 43.8 ± 8.0 nm for the wild-type strain and 55.0 ± 16.3 nm for the isolated mutant 2, Supplementary Material Figure S3. All five remaining isolated mutants were then tested at the stationary phase, and an increase in cell wall thickness was systematically observed with average values of 61.8 ± 14.3 nm, 48.2 ± 13.0 nm, 50.9 ± 8.3 nm, 47.5 ± 16.0 nm, and 47.0 ± 10.15 nm for the mutants 1, 3, 4, 5, and 6, respectively (Figure 5). This confirms the hypothesis that those mutations led to an increase in cell wall thickness. Multiple comparisons using one-way ANOVA revealed that all observed changes in cell wall thickness were statistically significant.

**Figure 6:**
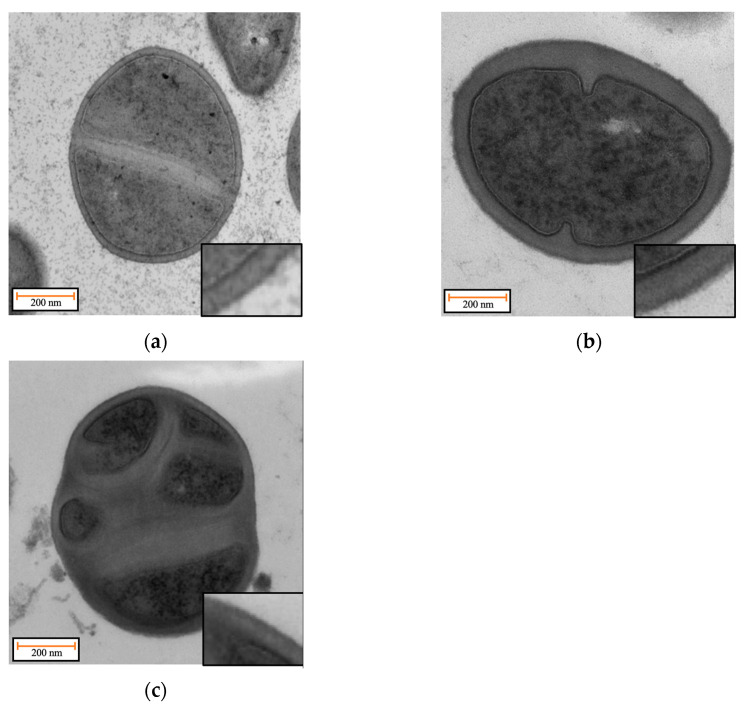
Transmission electron microscopy (TEM) images of *S. aureus* cells for investigating cellular characteristics: (**a**) depicts wild type cells during the stationary growth phase, displaying typical behaviour in terms of cell division and cell wall thickness; (**b**) shows TEM images of isolated mutant B cells, which exhibit increased cell wall thickness compared to the wild type cells; (**c**) highlights abnormal cell divisions in the mutant cells.

Extreme readings of cell wall thickness were also observed. Those are due to instances where the bacteria fail to divide correctly, leading to extra cell wall material. Figure 6 shows the difference between the wild-type strain with normal cell wall thickness and with the isolated mutant number 2, notably showing an image of an event where the cell fails to divide properly. Similar instances of failed cell divisions were observed in all isolated mutants, except for number 4, Supplementary Material Figure S1. This implies that this phenotype of increased cell wall thickening comes at the cost of events of division failure.

### 2.7. Changes in Gene Expression Levels Linked to Cell Wall Alterations

**Figure 7:**
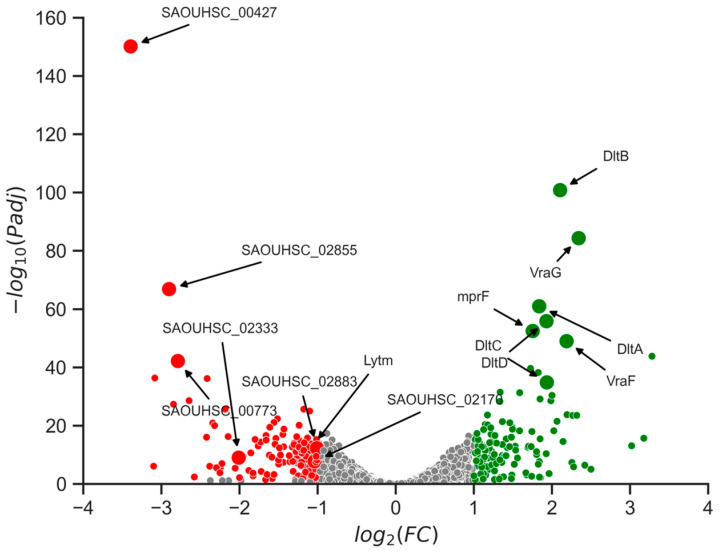
RNA-sequencing results for comparison of the wild type *S. aureus* and the isolated mutant 2. Data points are color-coded based on statistical significance and the magnitude of change in gene expression levels. Grey data points indicate non-significant changes. Red data points indicate significant changes with a high magnitude of expression change for genes which are downregulated, and green data points indicate significant changes with a high magnitude of expression change for genes which are upregulated.

In order to elucidate the mechanism by which current mutations impact the cell wall, an RNA sequencing analysis (RNA-seq) was performed on both the wild-type strain and one of the mutant strains. The selection of the mutant strain number 2 was based on its significant alteration of minimum inhibitory concentration (MIC) for vancomycin. The RNA-seq analysis revealed significant differential expression of 259 genes, with 130 upregulated and 129 downregulated genes (Figure 7). Subsequent analysis indicated that the observed changes in cell wall structure could be attributed to alterations in the expression of several of those genes.

RNA sequencing data revealed downregulation of genes involved in cell wall synthesis and maintenance. The *lytm* gene and SAOUHSC_02170 genes, coding for peptidoglycan hydrolases, were downregulated, leading to the hypothesis that observed cell wall phenotype could be attributed to a reduction of peptidoglycan hydrolysis [34]. SceD, a lytic transglycosylase with autolytic activity, and SAOUHSC_00427, a gene coding for an autolysin, were also downregulated [35]. Those could lead to cell division failure as autolysis is requiring for this process to occurs normally. Additionally, the genes SAOUHSC_00773, SAOUHSC_02855, and SAOUHSC_02883 coding for protein containing LysM domain, a protein motif for binding to peptidoglycans, were also downregulated [36].

The upregulation of genes such as *mprF* and *dltABCD* was observed, resulting in increased expression levels. Overexpression of *mprF* leads to the addition of lysine to the phosphatidylglycerol present in the cell membrane, while overexpression of *dltABCD* results in the addition of alanine to the teichoic acids on the cell wall. Both of these changes result in a less negatively charged membrane, thereby decreasing its sensitivity to cationic antimicrobial peptides [37,38]. Furthermore, downregulation of *vraF* and *vraG* was also observed. These genes are involved in the formation of a complex with the GraSR protein, leading to an increase in resistance to cationic antimicrobial peptides [39]. Those changes could explain a decrease in sensitivity to both bacteriocins as they are cationic peptides.

## 3. Discussion

### 3.1. The Study Findings

In the field, there is a growing consensus that bacteriocins with selective activities that take into account microbial interactions in the targeted microbiome represent a promising solution for the treatment of infections without further exacerbating antimicrobial resistance. However, the discovery of such compounds with the desired selectivity is challenging. Here, we showed that the use of the PARAGEN collection [20] led to the discovery of such bacteriocins for the treatment of *S. aureus* skin infections. Both the cerein 7B and cerein B4080 have the advantage of selectively inhibiting the growth of *S. aureus* without affecting the growth of the commensal skin bacteria, *S. epidermidis*.

Both cerein 7B and cerein B4080 are bacteriocins produced by *Bacillus* strains; specifically, *Bacillus cereus* Bc7 [21] for cerein 7B and *Bacillus cereus* B4080 for cerein B4080. These *Bacillus* strains are typically found in soil environments and are not commonly associated with the skin microbiome. As such, the observed selectivity of these bacteriocins against skin bacteria is unexpected.

Our findings suggest that cerein 7B anchors to the bacterial membrane through lysine and tryptophan residues, while its antimicrobial activity is dependent on GXXXG motifs and cysteines, which stabilize its alpha helix. However, the selectivity of the peptide between *S. aureus* and *S. epidermidis* remains unexplained. This selectivity could be due to differences in cell wall composition, surface charge, permeability, fluidity, and membrane stability of the bacteria. Alternatively, a membrane protein may serve as the target recognized by the bacteriocin.

There is a sense in previous studies [40] that bacteriocins are less likely to cause resistance than antibiotics. In our study, mutants that were able to grow in medium containing the bacteriocins were isolated and had an apparition rate of 1 for 1 million or 2 for a million. This indicates that the occurrence rate is higher in this case than what is typically observed, as the frequency of spontaneous mutations for antibiotic resistance is approximately 10^8^ to 10^9^ [41]. More concerning are the results obtained by an antibiogram performed at the Saint-Luc hospital in Brussels, which highlighted a decrease in sensitivity to vancomycin for some of the isolated mutants.

The development of resistance in *S. aureus* against antimicrobial peptides has already been observed and can involve two-component systems [42]. In the *aspRS* system, the presence of antimicrobial peptides is detected by the sensor, resulting in increased expression of genes such as *mprF* and *dltABCD*. Overexpression of these genes leads to changes in the cell membrane and cell wall, resulting in reduced sensitivity to cationic antimicrobial peptides [37,38]. Our study reveals a link between *walk/R* point mutations and the gene levels expression of *mprF* and *dltABCD*, likely to be responsible of sensitivity reduction to the selective bacteriocins discovered.

In our research, a WGS performed on those isolated mutants led to the observation that the two-component system *walK/R*, previously known to be involved in vancomycin resistance, was responsible for those changes and was involved in the resistance against both bacteriocins tested in this study. In our study, new mutations in *walK/R* that are causing a decrease in vancomycin sensitivity were discovered: the G223A and R376P in WalK; and the R222V and S105R in WalR. This information can help grow the current knowledge of this problematic resistance and improve diagnostics.

TEM analysis revealed that a thickening of the cell wall was present for all isolated mutants at the stationary phase, and that this phenotype led to occasional division failure. This is in line with previous work which proposed the peptidoglycan-clogging theory, which postulates that the passage of vancomycin molecules across thickened peptidoglycan layers is hindered and delayed, leading to resistance [27]. This phenotype of a thicker cell wall comes at the cost of division failure and therefore reduces fitness compared to the wild type in environments where no antimicrobial pressure is present. RNA sequencing results suggest that the observed cell wall thickening phenomenon may be attributed to the downregulation of peptidoglycan hydrolases. Furthermore, the failure in cell division could potentially be driven by the downregulation of autolysin.

### 3.2. Significance of the Findings—Implication for the Field

The findings of this study shed light on the potential and limitations of bacteriocins as an alternative to antibiotics. Despite their narrow spectrum of activity and lower propensity to induce resistance [28], our research has shown that cross-resistance can occur between bacteriocins and antibiotics. It is important to consider the implications of these findings for the development of selective antimicrobial compounds. Rather than looking for new compounds which theoretically do not induce any resistance; we suggest that selective compounds may be a more effective and sustainable approach. By targeting the pathogenic bacteria responsible for infections, commensal bacteria can be preserved, and the few pathogenic cells that have adapted to the selective pressure of the antimicrobial compounds would face harsh competition from commensal bacteria, likely leading to their elimination. This approach could pave the way for more effective and sustainable antimicrobial strategies in the future, ultimately resulting in fewer chronic infections and a lower spread of resistance.

### 3.3. Limitations of the Study

The present study has some limitations and weaknesses that need to be addressed. Firstly, a bacteriocin with selectivity between *S. aureus* and *S. epidermidis* is promising, but the complexity of the various skin microbiome interactions is not fully represented in our model. Further studies with a much broader representation of the skin microbiome should be conducted to better understand the impact of antimicrobial activity of bacteriocins on the skin microbiome. Secondly, while the DNA mutations in *S. aureus* isolated mutants and their effects on decreased sensitivity and changes in cell wall thickness were characterized, they were studied in groups. The individual introduction of those mutations one by one would be necessary to attribute them to the phenotype changes that were observed.

## 4. Materials and Methods

### 4.1. Spot Assay

To conduct the spot assay experiment, a solid M17 growth medium (37 g of M17 growth media and 10 g/L of glucose, and 1.5% agar; the final pH was adjusted to 6.9) is prepared as the first layer in a petri dish. The second layer is composed of the same M17 medium but containing a reduced agar content of 0.4% and is mixed with 100 microliters of an overnight preculture from the chosen indicator strain per 10 mL of the medium and poured onto the first layer. After laminar flow exposure for 20 min, a 2 microliter drop of 1 mg/mL cerein 7B or cerein B4080 solution is added at a specific location on the plate. The petri dish is then incubated overnight at 37 °C. An inhibition halo at the drop’s location indicates antimicrobial effectiveness, while its absence suggests a lack of activity. For *S. hominis* ATCC 27844, *S. epidermidis* ATCC 12228, *L. lactis* IL403, and *S. aureus* ATCC 6538, M17 grow medium was used. For *Propionibacterium acnes* ATCC 6919, the Reinforced Clostridial Agar (RCA) growth medium, composed of peptone (10 g/L), yeast extract (13 g/L), glucose 5 g/L), soluble starch (1 g/L), cysteine hydrochloride (0.5 g/L), sodium acetate (3 g/L), and sodium chloride (5 g/L), at adjusted pH 6.8, was used in anaerobic condition for culture growth.

### 4.2. Minimum Inhibition Concentraion (MIC)

The experimental procedure involved the preparation of a preculture of the indicator strain at a known concentration of 10^5^ CFU/mL. From this preculture, 37.5 microliters were transferred and mixed into 15 mL of growth liquid culture media. In a 96-well plate, 150 microliters of the indicator strain solution were dispensed into each well of a line, with the exception of the last column, where 200 microliters of liquid growth media were added. The total volume of each well was made up to 200 microliters by the addition of a solution containing the antimicrobial compound. A determined starting concentration of the antimicrobial compound was introduced in the first condition and was halved for each subsequent condition. This created a total of 11 conditions, with an additional condition added without any antimicrobial compound. Subsequent to the addition of the antimicrobial compound, the plate was incubated at 37 °C for 24 h. After the incubation period, the absorbance of each well was measured at 600 nm using a Hidex plate reader. To eliminate the absorbance value of the growth media alone, the values obtained in the last column, which served as a negative control with no bacterial growth, were subtracted from the values of the other wells in the same column. The resulting data for the remaining 11 wells in each column were plotted on a graph with the bacteriocin concentration on the *x*-axis and the normalized absorbance value on the *y*-axis. The EC50 value was calculated as the concentration of the antimicrobial compound at which 50% growth inhibition was achieved.

### 4.3. Structural Prediction Alpha Fold

In this research project, Alpha Fold [43], a deep learning-based method for predicting protein structure, was employed to predict the structures of various bacterial proteins. The specific implementation utilized was the “AlphaFold2-advanced.ipynb” notebook, as described in the publication by Jumper et al. [44]. The default parameters of the algorithm were employed, and the input provided to the program was the amino acid sequence of the target protein. Additionally, the homo oligomer value was set to 1.

### 4.4. In Vitro Production of Cerein 7B Bacteriocin and Mutated Versions

The DNA of the mutated version of the cerein 7B were made based on the method use in the PARAGEN collection [20]. The DNA template for the protein of interest was amplified and purified using standard molecular biology techniques. This DNA template was then produced using the Pure Express Protein Synthesis in vitro kit New England BioLabs, manual E6800 was utilized to assemble the reaction mixture, in accordance with the manufacturer’s protocol. The reagents and enzymes provided in the kit were combined in a specific ratio and order. The reaction mixture was then incubated at 37 °C for 2 h to facilitate the transcription and translation of the DNA template into the protein of interest. Following the incubation period, the synthesized protein was immediately utilized for the realization of spot assays.

### 4.5. Isolation of Mutants Able to Grow in Presence of Bacteriocins

The mutants were isolated on a petri dish plate made of M17 growth medium (37 g of M17 growth media and 10 g/L of glucose, and 1.5% agar; the final pH was adjusted to 6.9, and the cerein 7B bacteriocin at a 40 microgram/mL final concentration of the cerein B4080 at 200 microgram/mL final concentration). A pre-culture of *S. aureus* ATCC 6538 was grown in M17 liquid media. After overnight incubation at 37 °C, 50 microliters of the solution from this preculture were added onto the medium layer in the petri dish plate. The plates were then incubated overnight at 37 °C, and the number of colonies able to grow on the medium was counted once the incubation completed. Some colonies were grown in M17 liquid growth media, containing the bacteriocin at the same concentration for an overnight culture. The next day, they were stored at −80 °C with a glycerol concentration of 20%. The colony-forming units (CFUs) from the pre-culture of *S. aureus* ATCC 6538 was determined by serial dilution to determine the rate of resistance apparition.

### 4.6. S. aureus DNA Sequencing

The genomic DNA of *S. aureus* cells was extracted using the “GenElute™ Bacterial Genomic DNA Kit Protocol” from Merck. Following DNA extraction, the samples for the wild-type strain and the isolated mutants 1, 2, 7, and 8 underwent Illumina sequencing at Genewiz. Subsequently, the obtained DNA sequence data were analyzed for the presence of mutations in comparison to the wild-type strain *S. aureus* ATCC 6538 and the isolated mutants using the Galaxy platform. The data were uploaded to the Galaxy web-based platform and analyzed using the publicly available server at usegalaxy.org [45].

MinION sequencing was also used for the wild-type strain and the isolated mutants 3, 4, 5, and 6. The libraries for the *S. aureus* ATCC 6539 wild type DNA and the isolated mutant’s DNA were prepared using the “Rapid Barcoding Kit SQK-RBK004” provided by Oxford Nanopore Technology. The MinION Mk1B device, containing a flow cell R9, was used for this purpose. Base calling was performed using the Guppy software [46], while read filtering was conducted using Chopper with a minimum Q-score of 10. The genome alignment step was carried out using Minimap2 [47] followed by Samtools [48]. Single nucleotide polymorphism (SNP) calling was performed using VCFTools [49] and a custom Python script.

### 4.7. Vancomycin Antibiogram

The BD Phoenix automated system was employed to determine the antimicrobial susceptibility of the various bacterial strains using the PMIC-90 panel (Becton Dickinson, Sparks, MD, USA). The system utilizes the principle of broth microdilution to determine the minimum inhibitory concentrations (MIC) of vancomycin against the tested strains. The results were interpreted according to the Clinical and Laboratory Standards Institute (CLSI) guidelines.

### 4.8. Transmission Electronic Microscopy (TEM)

Bacteria samples were collected by centrifugation from liquid cultures, fixed for 1 h at room temperature in 2.5% glutaraldehyde in culture medium, rinsed in 0,1 M cacodylate buffer, and postfixed in 2% OsO4 in the same buffer. After serial dehydration in increasing ethanol concentrations, samples were embedded in agar 100 (Agar Scientific Ltd., Stansted, UK) and left to polymerize for 2 days at 60 °C. Ultrathin sections (50–70 nm thick) were collected in Formvar–carbon-coated copper grids by using a Leica EM UC6 ultramicrotome and stained with uranyl acetate and lead citrate by standard procedures. Observations were made on a Tecnai 10 transmission electron microscope (FEI). Morphometric analyses and images processing were performed using the SIS iTEM (Olympus) software. Cell wall length were measure on all cells presented in the images, for each cell, 4 measures were taken at each cardinal point. This methodology led to extreme reading when phenomenon of failed cell division was encountered.

### 4.9. RNA Sequencing and Analysis

The RNA extraction was realized on cell cultures once they reached the stationary growth phase by using the NucleoSpin RNA kit (Mascherey-Nagel). The cell lysis step was adapted, i.e., by using a lysosomes concentration 10 times higher than the one recommended in the kit. The quantity and quality of RNA extracted was measure by Qubit. The RNA sequencing was then performed by Seqalis.

The RNA-seq libraries were sequenced on an Illumina NextSeq 2000 system using 2 × 100 bp paired-end sequencing. The raw sequencing reads were pre-processed using Cutadapt v4.2 to remove adapter sequences and low-quality reads. The pre-processed reads were then aligned to the reference genome using STAR v2.7.10b with default parameters. Gene expression levels were estimated using featureCounts v2.0.3. The count data were imported into R using the DESeq2 package. Differential gene expression analysis was performed using DESeq2 with a false discovery rate (FDR) of less than 0.05 and an absolute log2-fold change greater than 1 considered significant. The differentially expressed genes were annotated using the Gene Ontology (GO) database and pathway analysis was performed using the Kyoto Encyclopedia of Genes and Genomes (KEGG) database.

## 5. Conclusions

In summary, this study provides valuable insights into the potential of bacteriocins as selective antimicrobial compounds for the treatment of *S. aureus* skin infections. The PARAGEN collection has facilitated the discovery of two bacteriocins, cerein 7B and cerein B4080, which exhibit selective inhibition against *S. aureus* while leaving commensal skin bacteria unaffected. Nevertheless, the study also highlights the possibility of cross-resistance between these bacteriocins and vancomycin, indicating the need for a cautious approach towards their clinical use as such use could worsen the current problem of vancomycin resistance in *S. aureus*. Furthermore, the identification of new mutations in the *walK/R* two-component system, which cause decreased vancomycin sensitivity and cell wall thickening, represents a significant contribution to our understanding of problematic resistance and offers new diagnostic possibilities. The findings of this study have crucial implications for the development of more effective and sustainable antimicrobial strategies, which could lead to a significant reduction in chronic infections and a decrease in the spread of resistance.

## 6. Patents

The authors would like to acknowledge that the results presented in this publication have led to the filing of two patent applications: [WO 2022/253741 A1—Cerein 7B bacteriocin for new application] and [WO 2022/253743 A1—Bacteriocin for new application].

## Figures and Tables

**Table 1:** Activity spectrum of the cerein 7B and cerein B4080 on various bacterial strains.

Bacterial Species	Cerein 7B	Cerein B4080
*S. aureus* ATCC 6538	Active	Active
*S. epidermidis* ATCC 12228	Non active	Non active
*S. hominis* ATCC 27844	Non active	Active
*Propionibacterium acnes* ATCC 6919	Non active	Non active
*Escherichia coli* MG 1655	Non active	Non active
*Lactococcus lactis* IL1403	Active	Active

**Table 2:** List of DNA mutations found in isolated *S. aureus* mutants after exposure to cerein 7B and cerein B4080′s selective pressure.

Isolated Mutant	Mutation Position	Mutation	Amino Acid Mutation	Protein Affected
1	26033	t-g	I303S	WalK
	26252	g-c	R376P	WalK
	1621507	g-t	H228N	AroE
2	26033	t-g	I303S	WalK
	25075	c-t	R222V	WalR
	1621507	g-t	H228N	AroE
3	24743	c-t	A111V	WalR
	116151	c-a	Y446Q	Hypothetical DNA-binding protein
	1329116	a-t	K385I	Tkt
4	26033	t-g	I303S	WalK
	233744	c-a	Y339T	NikA
	995921	g-a	V304M	MenD
	1621507	g-t	H228N	AroE
5	26033	t-g	I303S	WalK
	1621507	g-t	H228N	AroE
6	25793	g-c	G223A	WalK
	26033	t-g	I303S	WalK
	1621507	g-t	H228N	AroE
7	26033	t-g	I303S	WalK
	26272	g-a	V383I	WalK
	1621507	g-t	H228N	AroE
	407356	g-t	V196L	SSL5-like
	241630	c-t	H12Y	Hypothetical alcohol dehydrogenase
8	24724	a-c	S105R	WalR
	27103	g-c	A55P	Yych
	1329116	a-t	K385I	Tkt

**Table 3:** Vancomycin sensitivity of the wild type *S. aureus* and the isolated mutants.

Bacterial Species	Vancomycin MIC (µg/mL)
*S. aureus* ATCC 6538	≤0.5
Isolated mutant 1	1
Isolated mutant 2	2
Isolated mutant 3	≤0.5
Isolated mutant 4	≤0.5
Isolated mutant 5	≤0.5
Isolated mutant 6	1
Isolated mutant 7	1
Isolated mutant 8	1

## Data Availability

The datasets used and analysed during the current study are available from the corresponding author upon reasonable request.

## Supplementary Materials

The following supporting information can be downloaded at: https://www.mdpi.com/article/10.3390/antibiotics12060947/s1, Figure S1: Transmission Electron Microscopy (TEM) Additional images; Figure S2: Spot assays of alanine scanning; Figure S3: Transmission Electron Microscopy comparison stationary phase with exponential phase. Table S1: Alanine scanning of cerein 7B—mutation list and spot assays results; Table S2: DNA differences in between *S. aureus* ATCC 6538 used in this study and its reference genome (NZ_CP20020).
