# Supplementary material for: Selective Bacteriocins: A Promising Treatment for Staphylococcus aureus Skin Infections Reveals Insights into Resistant Mutants, Vancomycin Resistance, and Cell Wall Alterations
Source: Antibiotics (Basel). 2023 May 23;12(6):947. doi: 10.3390/antibiotics12060947 (PMC10295673; doi:10.3390/antibiotics12060947)
Supplement: Supplementary file 1 [file antibiotics-12-00947-s001.zip › antibiotics-2397877-supplementary.pdf]

Supplementary materials

Transmission Electron Microscopy (TEM) Additional images

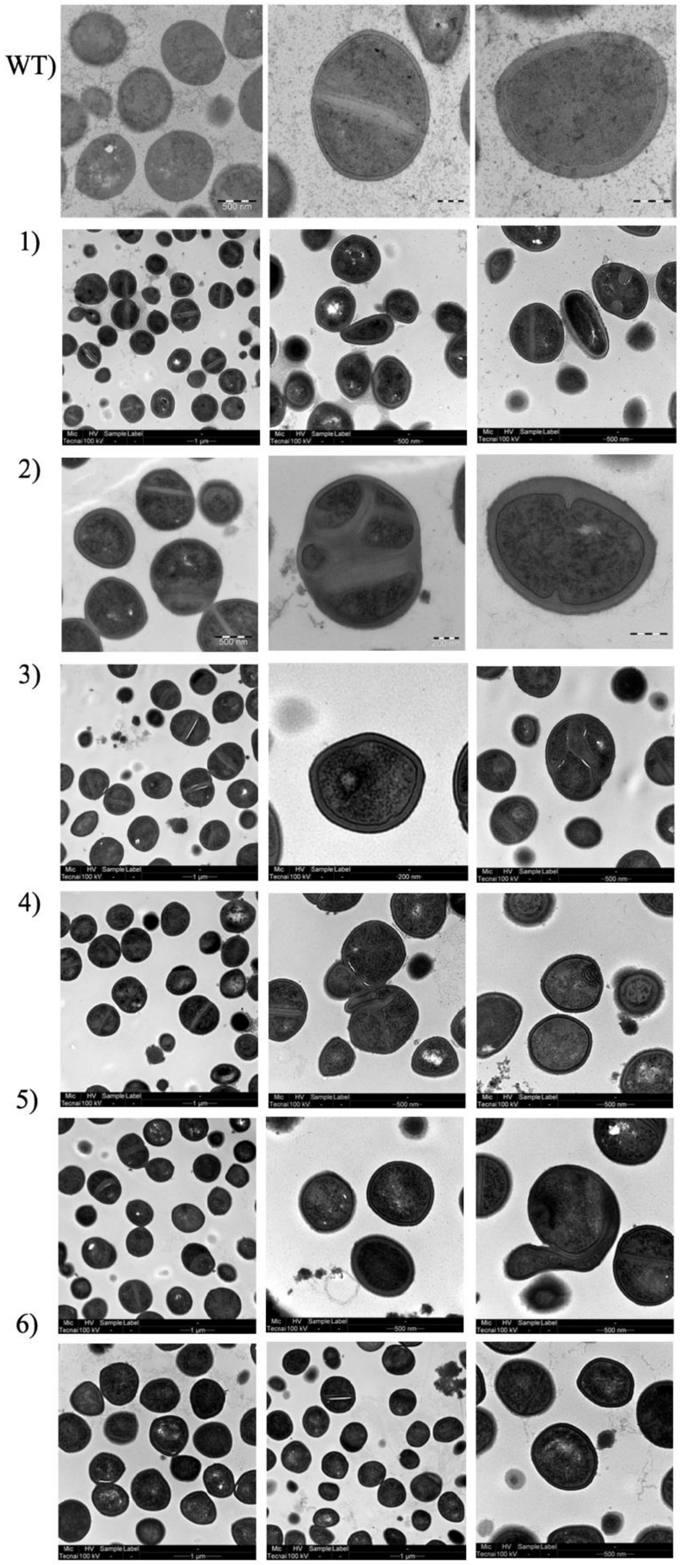

Figure S1: Supplementary TEM images of the wild-type S. aureus ATCC 6538 strain and six mutant strains resistant to Cerein 7B selective pressure. Some of the images reveal aberrant cell division events in the mutant strains

Cerein 7B alanine scanning

Table S1, Alanine scanning of cerein 7B – mutation list and spot assays results

**Bacteriocin**	**Plate**	**Amino acid sequence**	***L. lactis***	***S. aureus***
WT	A1	GWWNSWGKCVAGTIGGAGTGGLGGAAAGSAVPVIGTGIGGAIGGVSGGLTGAATFC	(+)	(+)
Cerein 7B-G1A	A2	AWWNSWGKCVAGTIGGAGTGGLGGAAAGSAVPVIGTGIGGAIGGVSGGLTGAATFC	(+)	-
Cerein 7B-W2A	A3	GAWNSWGKCVAGTIGGAGTGGLGGAAAGSAVPVIGTGIGGAIGGVSGGLTGAATFC	-	-
Cerein 7B-W3A	A4	GWANSWGKCVAGTIGGAGTGGLGGAAAGSAVPVIGTGIGGAIGGVSGGLTGAATFC	-	-
Cerein 7B-N4A	A5	GWWASWGKCVAGTIGGAGTGGLGGAAAGSAVPVIGTGIGGAIGGVSGGLTGAATFC	(+)	(+)
Cerein 7B-S5A	A6	GWWNAWGKCVAGTIGGAGTGGLGGAAAGSAVPVIGTGIGGAIGGVSGGLTGAATFC	(+)	(+)
Cerein 7B-W6A	A7	GWWNSAGKCVAGTIGGAGTGGLGGAAAGSAVPVIGTGIGGAIGGVSGGLTGAATFC	-	-
Cerein 7B-G7A	A8	GWWNSWAKCVAGTIGGAGTGGLGGAAAGSAVPVIGTGIGGAIGGVSGGLTGAATFC	(+)	-
Cerein 7B-K8A	A9	GWWNSWGACVAGTIGGAGTGGLGGAAAGSAVPVIGTGIGGAIGGVSGGLTGAATFC	-	-
Cerein 7B-C9A	A10	GWWNSWGKAVAGTIGGAGTGGLGGAAAGSAVPVIGTGIGGAIGGVSGGLTGAATFC	-	-
Cerein 7B-V10A	A11	GWWNSWGKCAAGTIGGAGTGGLGGAAAGSAVPVIGTGIGGAIGGVSGGLTGAATFC	-	-
Cerein 7B-G12A	A12	GWWNSWGKCVAATIGGAGTGGLGGAAAGSAVPVIGTGIGGAIGGVSGGLTGAATFC	(+)	-
Cerein 7B-G13A	B1	GWWNSWGKCVAGAIGGAGTGGLGGAAAGSAVPVIGTGIGGAIGGVSGGLTGAATFC	(+)	(+)
Cerein 7B-I14A	B2	GWWNSWGKCVAGTAGGAGTGGLGGAAAGSAVPVIGTGIGGAIGGVSGGLTGAATFC	(+)	-
Cerein 7B-G15A	B3	GWWNSWGKCVAGTIAGAGTGGLGGAAAGSAVPVIGTGIGGAIGGVSGGLTGAATFC	(+)	-
Cerein 7B-G16A	B4	GWWNSWGKCVAGTIGAAGTGGLGGAAAGSAVPVIGTGIGGAIGGVSGGLTGAATFC	(+)	(+)
Cerein 7B-G18A	B5	GWWNSWGKCVAGTIGGAATGGLGGAAAGSAVPVIGTGIGGAIGGVSGGLTGAATFC	(+)	(+)
Cerein 7B-T19A	B6	GWWNSWGKCVAGTIGGAGAGGLGGAAAGSAVPVIGTGIGGAIGGVSGGLTGAATFC	(+)	-
Cerein 7B-G20A	B7	GWWNSWGKCVAGTIGGAGTAGLGGAAAGSAVPVIGTGIGGAIGGVSGGLTGAATFC	(+)	-
Cerein 7B-G21A	B8	GWWNSWGKCVAGTIGGAGTGALGGAAAGSAVPVIGTGIGGAIGGVSGGLTGAATFC	(+)	(+)
Cerein 7B-L22A	B9	GWWNSWGKCVAGTIGGAGTGGAGGAAAGSAVPVIGTGIGGAIGGVSGGLTGAATFC	-	-
Cerein 7B-G23A	B10	GWWNSWGKCVAGTIGGAGTGGLAGAAAGSAVPVIGTGIGGAIGGVSGGLTGAATFC	(+)	(+)
Cerein 7B-G24A	B11	GWWNSWGKCVAGTIGGAGTGGLGAAAAGSAVPVIGTGIGGAIGGVSGGLTGAATFC	-	-
Cerein 7B-G28A	B12	GWWNSWGKCVAGTIGGAGTGGLGGAAAASAVPVIGTGIGGAIGGVSGGLTGAATFC	-	-
Cerein 7B-S29A	C1	GWWNSWGKCVAGTIGGAGTGGLGGAAAGAAVPVIGTGIGGAIGGVSGGLTGAATFC	(+)	-
Cerein 7B-V31A	C2	GWWNSWGKCVAGTIGGAGTGGLGGAAAGSAAPVIGTGIGGAIGGVSGGLTGAATFC	(+)	-
Cerein 7B-P32A	C3	GWWNSWGKCVAGTIGGAGTGGLGGAAAGSAVAVIGTGIGGAIGGVSGGLTGAATFC	(+)	-
Cerein 7B-V33A	C4	GWWNSWGKCVAGTIGGAGTGGLGGAAAGSAVPAIGTGIGGAIGGVSGGLTGAATFC	(+)	-
Cerein 7B-I34A	C5	GWWNSWGKCVAGTIGGAGTGGLGGAAAGSAVPVAGTGIGGAIGGVSGGLTGAATFC	-	-
Cerein 7B-G35A	C6	GWWNSWGKCVAGTIGGAGTGGLGGAAAGSAVPVIATGIGGAIGGVSGGLTGAATFC	-	-
Cerein 7B-T36A	C7	GWWNSWGKCVAGTIGGAGTGGLGGAAAGSAVPVIGAGIGGAIGGVSGGLTGAATFC	(+)	
Cerein 7B-G37A	C8	GWWNSWGKCVAGTIGGAGTGGLGGAAAGSAVPVIGTAIGGAIGGVSGGLTGAATFC	(+)	(+)
Cerein 7B-I38A	C9	GWWNSWGKCVAGTIGGAGTGGLGGAAAGSAVPVIGTGAGGAIGGVSGGLTGAATFC	-	-
Cerein 7B-G39A	C10	GWWNSWGKCVAGTIGGAGTGGLGGAAAGSAVPVIGTGIAGAIGGVSGGLTGAATFC	(+)	-
Cerein 7B-G40A	C11	GWWNSWGKCVAGTIGGAGTGGLGGAAAGSAVPVIGTGIGAAIGGVSGGLTGAATFC	(+)	(+)
Cerein 7B-I42A	C12	GWWNSWGKCVAGTIGGAGTGGLGGAAAGSAVPVIGTGIGGAAGGVSGGLTGAATFC	-	-
Cerein 7B-G43A	D1	GWWNSWGKCVAGTIGGAGTGGLGGAAAGSAVPVIGTGIGGAIAGVSGGLTGAATFC	-	-
Cerein 7B-G44A	D2	GWWNSWGKCVAGTIGGAGTGGLGGAAAGSAVPVIGTGIGGAIGAVSGGLTGAATFC	(+)	(+)
Cerein 7B-V45A	D3	GWWNSWGKCVAGTIGGAGTGGLGGAAAGSAVPVIGTGIGGAIGGASGGLTGAATFC	(+)	
Cerein 7B-S46A	D4	GWWNSWGKCVAGTIGGAGTGGLGGAAAGSAVPVIGTGIGGAIGGVAGGLTGAATFC	(+)	(+)
Cerein 7B-G47A	D5	GWWNSWGKCVAGTIGGAGTGGLGGAAAGSAVPVIGTGIGGAIGGVSAGLTGAATFC	-	-
Cerein 7B-G48A	D6	GWWNSWGKCVAGTIGGAGTGGLGGAAAGSAVPVIGTGIGGAIGGVSGALTGAATFC	(+)	(+)
Cerein 7B-L49A	D7	GWWNSWGKCVAGTIGGAGTGGLGGAAAGSAVPVIGTGIGGAIGGVSGGATGAATFC	-	-
Cerein 7B-T50A	D8	GWWNSWGKCVAGTIGGAGTGGLGGAAAGSAVPVIGTGIGGAIGGVSGGLAGAATFC	(+)	-
Cerein 7B-G51A	D9	GWWNSWGKCVAGTIGGAGTGGLGGAAAGSAVPVIGTGIGGAIGGVSGGLTAAATFC	(+)	(+)
Cerein 7B-T54A	D10	GWWNSWGKCVAGTIGGAGTGGLGGAAAGSAVPVIGTGIGGAIGGVSGGLTGAAAFC	(+)	(+)
Cerein 7B-F55A	D11	GWWNSWGKCVAGTIGGAGTGGLGGAAAGSAVPVIGTGIGGAIGGVSGGLTGAATAC	-	-
Cerein 7B-C56A	D12	GWWNSWGKCVAGTIGGAGTGGLGGAAAGSAVPVIGTGIGGAIGGVSGGLTGAATFA	-	-

"(+)" represent an activity, the presence of an inhibition halo on the plate, while "-" represents no activity, the absence of an inhibition halo on the plate.

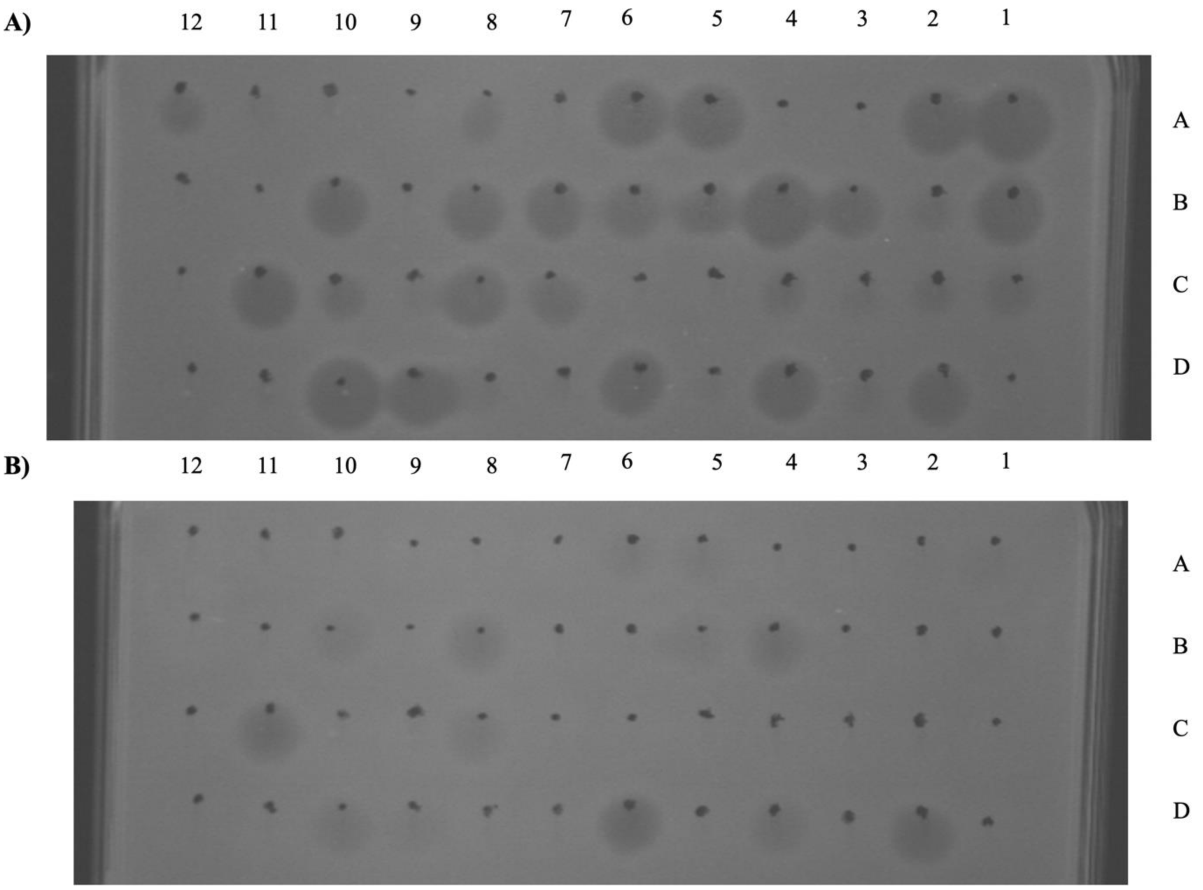

Figure S2: (A,B) displays the spot assays of the 48 bacteriocins that were produced in vitro, with the positions of the wild-type and mutant variants indicated in Table 1. Inhibition halos are observed in regions where the mutated versions of bacteriocins exhibited activity.

Differences in between the S. aureus ATCC 6538 used in this study and its reference genome (NZ_CP20020)

Comparison between the reference genome (NZ_CP020020) and the DNA sequencing data obtained on the *S. aureus ATCC 6538* strain used in this research

POS	TYPE	REF	ALT	FTYPE	STRAND	NT_POS	AA_POS	EFFECT	LOCUS_TAG	GENE
55146	snp	G	A	CDS	+	145/483	49/160	missense_variant c.145G>A p.Asp49Asn	B4602_RS00250	
109242	snp	T	C	CDS	+	254/663	85/220	missense_variant c.254T>C p.Val85Ala	B4602_RS00495	deoC
266876	snp	T	G	CDS	+	802/1437	268/478	missense_variant c.802T>G p.Tyr268Asp	B4602_RS01165	
315858	snp	T	G	CDS	+	303/924	101/307	missense_variant c.303T>G p.Ile101Met	B4602_RS01440	
574451	snp	A	T	CDS	+	1671/2826	557/941	missense_variant c.1671A>T p.Arg557Ser	B4602_RS02790	sdrC
777056	snp	T	C	CDS	+	19/573	7/190	missense_variant c.19T>C p.Tyr7His	B4602_RS03865	raiA
936739	snp	A	G	CDS	+	214/927	72/308	missense_variant c.214A>G p.Ser72Gly	B4602_RS04750	
975730	snp	T	C	CDS	+	118/2325	40/774	stop_lost c.118T>C p.Ter40Glnext*?	B4602_RS04930	
1008961	mnp	TT	GC	CDS	-	305/435	102/144	missense_variant c.304_305delAAinsGC p.Asn102Ala	B4602_RS05115	
1092692	snp	G	C	CDS	+	235/324	79/107	missense_variant c.235G>C p.Asp79His	B4602_RS05565	isdG
1098197	ins	A	AT	CDS	-	921/927	307/308	frameshift_variant c.921dupA p.Phe308fs	B4602_RS05595	
1205505	snp	A	G	CDS	-	2562/2607	854/868	synonymous_variant c.2562T>C p.Gly854Gly	B4602_RS06140	yfhO
1413666	snp	T	G	CDS	-	492/660	164/219	missense_variant c.492A>C p.Lys164Asn	B4602_RS07190	arlR
1482826	snp	A	G	CDS	-	322/1293	108/430	missense_variant c.322T>C p.Tyr108His	B4602_RS07400	asnS
2052278	snp	C	T	CDS	+	662/744	221/247	missense_variant c.662C>T p.Ala221Val	B4602_RS10625	
2313648	snp	A	G	CDS	+	617/690	206/229	missense_variant c.617A>G p.Asp206Gly	B4602_RS12055	
2382502	mnp	AT	CA							

Table S2, presents a comparison between the reference genome (NZ_CP020020) and the DNA sequencing data generated from the S. aureus ATCC 6538 strain utilized in this study. The table includes detailed information regarding the position of each mutation, its type, the corresponding DNA mutation, the nucleotide position within the coding region, the amino acid position in the protein sequence, the mutation's effect, the locus-tag where the mutation is localized, and the corresponding gene affected if annotated in the reference genome.

Transmission Electron Microscopy comparison stationary phase with exponential phase

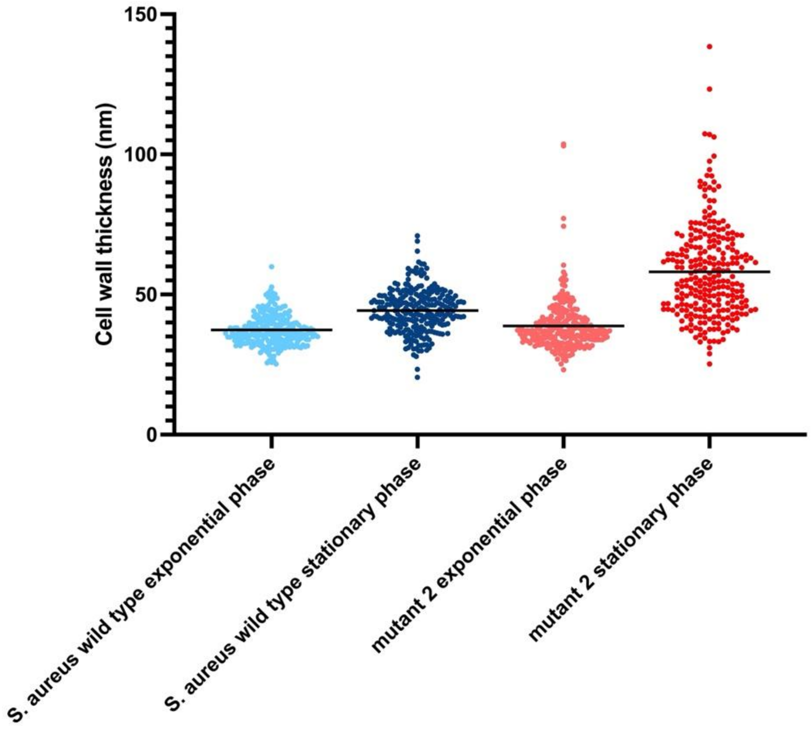

Figure S3 presents the results of transmission electron microscopy (TEM) measurement of the cell wall thickness in exponential phase and in the stationary phase for both the wild-type strain of *Staphylococcus aureus* ATCC 6538 and the isolated mutant 2. The TEM images provide insight into the comparison of the cell wall thickness between the two strains, offering valuable information about the cell wall thickness in various growth stages.
